# Trophic Niche Differentiation in Rodents and Marsupials Revealed by Stable Isotopes

**DOI:** 10.1371/journal.pone.0152494

**Published:** 2016-04-06

**Authors:** Mauro Galetti, Raisa Reis Rodarte, Carolina Lima Neves, Marcelo Moreira, Raul Costa-Pereira

**Affiliations:** 1 Departamento de Ecologia, Universidade Estadual Paulista (UNESP), C.P. 199, 13506–900 Rio Claro, São Paulo, Brazil; 2 Casa da Floresta Assessoria Ambiental Ltda., 13415–030, Piracicaba, São Paulo, Brazil; 3 CENA, Universidade de São Paulo, 13416–903, Piracicaba, São Paulo, Brazil; 4 Programa de Pós-graduação em Ecologia e Biodiversidade, Universidade Estadual Paulista (UNESP), C.P. 199, 13506–900 Rio Claro, São Paulo, Brazil; University of Sydney, AUSTRALIA

## Abstract

Tropical rainforests support the greatest diversity of small mammals in the world, yet we have little understanding about the mechanisms that promote the coexistence of species. Diet partitioning can favor coexistence by lessening competition, and interspecific differences in body size and habitat use are usually proposed to be associated with trophic divergence. However, the use of classic dietary methods (e.g. stomach contents) is challenging in small mammals, particularly in community-level studies, thus we used stable isotopes (*δ*^13^C and *δ*^15^N) to infer about trophic niche. We investigated i) how trophic niche is partitioned among rodent and marsupial species in three Atlantic forest sites and ii) if interspecific body size and locomotor habit inequalities can constitute mechanisms underlying the isotopic niche partitioning. We found that rodents occupied a broad isotopic niche space with species distributed in different trophic levels and relying on diverse basal carbon sources (C3 and C4 plants). Surprisingly, on the other hand, marsupials showed a narrow isotopic niche, both in *δ*^13^C and *δ*^15^N dimensions, which is partially overlapped with rodents, contradicting their description as omnivores and generalists proposed classic dietary studies. Although body mass differences did not explained the divergence in isotopic values among species, groups of species with different locomotor habit presented clear differences in the position of the isotopic niche space, indicating that the use of different forest strata can favor trophic niche partitioning in small mammals communities. We suggest that anthropogenic impacts, such as habitat modification (logging, harvesting), can simplify the vertical structure of ecosystems and collapse the diversity of basal resources, which might affect negatively small mammals communities in Atlantic forests.

## Introduction

How different species can coexist in a community has long being a central issue in ecology and various theories have been proposed to explain such phenomenon [1–4]. Niche-based approaches postulates that co-occurring species are required to exhibit ecological differences in at least one niche dimension, such as space use [5] or food preferences [6]. In other words, niche theory predicts that two species cannot share exactly the same niche; otherwise, one must be excluded by competition, which constrains the number of coexisting species in biological communities [7]. Indeed, many examples of niche differentiation between co-occurring species have supported this idea in diverse taxonomic groups [8]. However, understand how so many species partition resources and the underlying mechanisms allowing coexistence in highly diverse tropical communities is still an open issue in modern ecology, which has challenged ecologists for more than half a century [1].

Nutritional or trophic dimension is the most explored niche component in ecological studies [8]. As animals acquire and assimilate resources for growth and reproduction, trophic niche dimension is directly related to the average fitness of organisms [9]. Therefore, trophic partitioning between co-occurring species can favor coexistence via lessening the niche overlap and the intensity of interspecific competition [10]. Diet partitioning can arise via many types of ecological differences between species, but studies have focused mainly in interspecific differences in functional traits and space use among community members [8].

Body size is a key trait in ecological interactions, and can be a surrogate of resource use and energetics for many animal groups. Size is directly related of how organisms experience the environment, impacting on its fitness and performance [11]. In this line, the spatial scaling law postulates that interspecific differences in size will result in sufficiently distinct niches that allow the persistence of competitors [12]. For instance, body size can constraint the type and size of food resources that can be explored (e.g. gape limitation). Hence, divergences in body size among community members can constitute an underlying mechanism of niche partitioning [13, 14]. In turn, the spatial arrangement of species also can lead to niche divergence (e.g. use of different vertical strata in forests). Studies in diverse taxonomic groups have shown that even subtle differences in foraging microhabitats between sympatric species can potentially buffer competition intensity and favor species’ coexistence [15].

Small mammals are promising study models to investigate trophic relations in tropical communities because they constitute a diverse group, with conspicuous interspecific variations in body size and microhabitat use, and consume a broad range of food resources [16]. The Atlantic forests in South America holds one of the greatest diversity of small mammals in the Neotropics, harboring more than 120 species of rodents and marsupials [17], with up to 23 species living sympatric [18]. Information on the diet of rodents and marsupials are often obtained through direct observations of feeding activity in the field [19], analysis of fecal samples [20], gut content [21] or captive feeding trials [22, 23]. However these direct methods may not accurately determine diets because they only reveal undigested food remains or constitute temporally punctual samples of the consumed resources [24]. Moreover, direct observations on the diet of small mammals are difficult in forested habitats because of their small size and the nocturnal activity. One alternative that has been increasingly applied to infer food sources [25] and discriminate trophic niches in ecological studies is the use of stable carbon and nitrogen isotopes [16, 26, 27]. Stable isotopes provide information about the assimilated food on different time scales ranging from hours to decades depending on the tissue analyzed [28]. Hard tissues (e.g. hair) accumulate information on diet over a long period, probably several weeks and months [29].

Here, we aim to understand the trophic relationships in community-rich species of small mammals in the Brazilian Atlantic forest using stable isotopes ratios (*δ*^13^C and *δ*^15^N). First, we compared the size and degree of overlap in the isotopic niche space between rodents and marsupials. If diet divergence is an important mechanism to promote coexistence of these groups in tropical forests, we expect a low isotope niche space overlap between rodents and marsupials. Additionally, based on the long-standing idea that niche partitioning in tropical small mammal related species is a result of differential use of vertical strata and contrasts in body size [30–34], we hypothesized that interspecific differences in locomotor habits and body mass can be mechanisms underlying the isotopic niche partitioning in Atlantic rainforest communities. Accordingly, we expect that i) there is isotopic niche segregation between groups of species differing in locomotor habits; and ii) species-pairs with similar body mass have more similar isotopic composition than species-pairs with large body mass differences.

## Materials and Methods

### Study site

**Fig 1:**
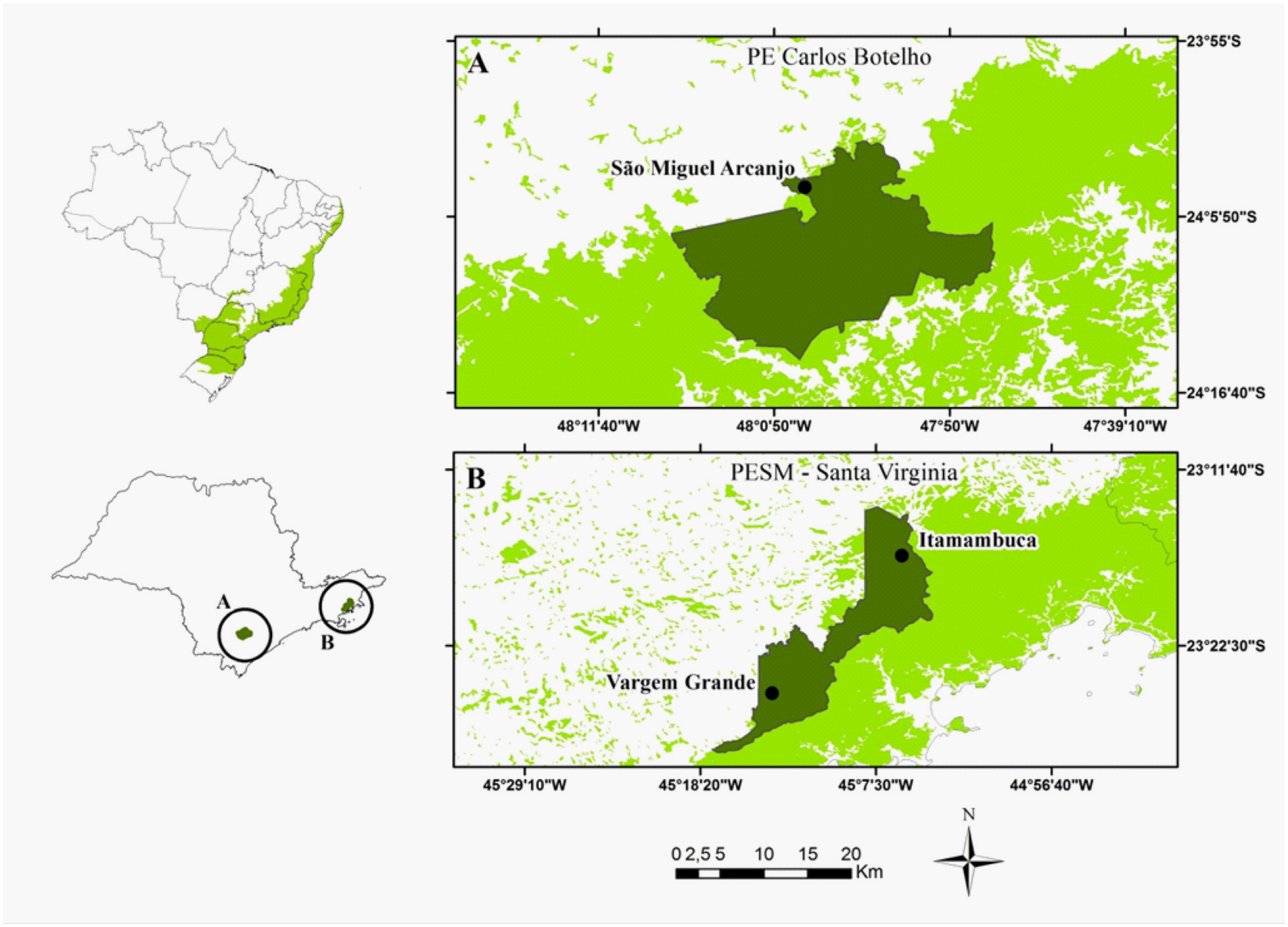
Location of the three studied Atlantic forest areas in São Paulo, Brazil. (A) Carlos Botelho State Park (in dark green) and the upper part of the park, São Miguel Arcanjo field-station evidenced by a black dot, (B) Santa Virginia nucleus (in dark green) and their both field-station (Itamambuca e Vargem Grande) indicated by black dots. In light green we have the current distribution of the Serra do Mar massif in the Brazilian Atlantic Forest.

This study was carried out in three sites in the Serra do Mar massif, the largest remnant of continuous Atlantic forest with an area of 1,200,000 ha [35]: Itamambuca (hereafter ITA, 45°5’W/23°19’S) and Vargem Grande basis (hereafter VG, 45°14’W/23°26’S) both in Serra do Mar State Park and São Miguel Arcanjo basis in Carlos Botelho State Park (hereafter CB, 48°06’W/24°13’S), south-east Brazil (Fig 1). Sites have similar altitudes (ranging from 700 to 1,100 meters) and the same vegetation type (Montane Atlantic Rainforest) [36]. Although similar in many ecological aspects, these three sites differ in the abundance of large mammals that may interfere in the diet of small mammals [37]. At least 25 species, nine marsupials and 16 small rodents, compose the small mammal communities of this region [38, 39].

### Sampling of small mammals and food resources

The small mammal sampling took place bimonthly throughout one year (VG and ITA between 2010 to 2011 and CB between 2011 to 2012), totaling six field trips equally distributed in dry and wet seasons per area. During each field trip, we trapped five consecutive nights. To optimize our sampling we used both live traps and pitfall traps. In each area we set up three grids with live-traps and six transects of pitfalls. Each grid covered an area of 0.6 ha and had live-traps of two types and four sizes, Sherman (small, 25 x 7.5 x 9.5 cm; medium, 30 x 7.5 x 9.5 cm; large, 37.5 x 10 x 12 cm; H.B. Sherman Trap, Inc., Tallahassee, Florida, USA) and Tomahawk (45 x 16 x 16 cm). In each grid, there were 24 capture stations. Each station had one Sherman trap (a small, a medium or a large, randomly chosen). Furthermore, six stations also had a Tomahawk trap. In total, there were 90 live-traps (30 traps per grid). Every two pitfall-trap transects were placed parallel to each other and 30-m apart. Each line had four plastic buckets (60 liters, with 40 cm of top diameter, 35 cm of bottom diameter, and 56-cm deep), buried with the rim at the ground level. In each line, buckets were buried every 10 m and connected to each other with 0.5-m tall drift fence that extended an additional 10 m at each end, totaling 50 m of fence. In total, we used 24 buckets. To minimize pseudo-replication at the grid level we spaced all grids and transects in each site at least 200 m from each other based on distances moved per night by Atlantic Forest small mammals [40]. Overall, our sampling effort consisted of 3420 trap-nights, being 2700 for live-traps and 720 for pitfall-traps per site.

We baited all traps including pitfall-traps with a mixture of bacon, corn meal, peanut butter and mashed bananas, checked them each morning and re-baited if necessary. We used bait in buckets in order to minimize starvation of the animals caught, which could spend more than 12 hours inside the buckets before released. All individuals captured were marked with a numbered ear tag (Ear Tags, National Band and Tag Co., Newport, Kentucky, USA), identified, weighed and measured. We collected hair samples for the stable isotope analysis and then released the animal at the same spot where trapped [37].

In case of identification's uncertainty, the animal was collected and carried to Instituto Butantã for cytogenetic analysis [41]. The animals were deposited as reference material in vertebrate scientific collection of Laboratório de Mastozoologia e Biogeografia, Universidade Federal do Espírito Santo, ES, Brazil. The collecting permits were provided by Instituto Brasileiro do Meio Ambiente e dos Recursos Naturais Renováveis (IBAMA #14428–2, IBAMA #31941–1) and Fundação Florestal do Estado de São Paulo allowed the field work at the three Protected Areas cited above (#347/2011 D29/11).

Potential food resource samples were visually found and manually collected randomly and concomitantly to small mammals near or inside the trapping grids and along the pitfall lines for a stable isotope habitat baseline. In an attempt to identify the maximum resource availability at the trapping sites, we sampled food items that were recorded in previous studies on diet of rodents and marsupials in Atlantic forest, such as plant tissues (fruits and seeds), invertebrates and fungi [21, 42].

### Stable isotope analysis

Prior to the analysis, all food samples were oven-dried at 40°C to remove traces of water and subsequently cut into fine pieces for analysis. The concentrations of nitrogen and carbon isotopes in mammals’ hair and food resources were determined by Continuous Flow Isotope Ratio Mass Spectrometry, using a Thermo Delta Plus mass spectrometer (Bremen, Germany) coupled to a Carlo Erba CHN 1110 elemental analyzer (Milan, Italy). The ^13^C/^12^C and ^15^N/^14^N isotope ratios were evaluated and compared to calibrated gas ratios using Pee Dee Belemnite carbonate and atmospheric N_2_, respectively according to the formula:

$$\delta X=[\left(\frac{R\;sampled}{R\;standard}\right)-1]$$

where X refers to ^13^C or ^15^N and R_sample_ and R_standard_ are the ^13^C/^12^C or ^15^N/^14^N ratios of sample and standard, respectively. The results were defined in delta notation (*δ*) and reported as deviations in parts per mil (‰) in relation to international patterns. Precision was estimated by the SD (standard deviations) of 44 replicates of an internal standard along the sample analyses as 0.09 and 0.12‰ for C and N, respectively.

Here, we use isotopic values of small mammals to represent the assimilated food resources and, then, infer about trophic niche of organisms. The ratio ^13^C/^12^C differ between plants with distinct metabolisms and can propagate through the food chains. In general, C3 plants have *δ*^13^C between -33 and -24 ‰, C4 plants between -16 and -10 ‰ and CAM species between -20 to -10 ‰. Thus, based on the position of a given consumer along *δ*^13^C axis is possible to discriminate individuals, species or groups with distinct assimilated resources. In turn, the ratio of ^15^N/^14^N tends to increase in food chain with trophic level [43].

All isotope analyses were performed in Isotope Ecology Laboratory, Centro de Energia Nuclear e Agricultura (CENA) at Universidade de São Paulo, Piracicaba. Considering to our studied group, i.e adjusted consumer isotope values using the most appropriate mean diet-to-tissue trophic enrichment values 2.7‰ for *δ*^15^N and 2.4‰ for *δ*^13^C [44, 45]. Details on the results are in S1 Table.

### Data analysis

Prior to carbon (*δ*^13^C) and nitrogen (*δ*^15^N) analysis, we checked if stable isotope values are normally distributed using Shapiro–Wilk test. To compare the isotopic niche space between rodents and marsupials we calculate standard ellipses, which represent the isotopic niche size of roughly 40% of species within the groups using bivariate normal distributions [46]. This method accounts for core isotopic niche areas, being less sensitive to sample size than convex hull methods, allowing more robust comparisons among groups [47]. To control sample sizes, we used the corrected version of the standard ellipse area (SEA_*C*_) [48]. To compare isotopic niche space between groups we compute the Bayesian estimate of the standard ellipse area (SEA_*B*_) [49]. We calculated and compared ellipses in each sampled community and for all sites polled together (using all captured individuals).

To evaluate if interspecific variations in microhabitat use are associated with isotopic niche partitioning we first classified the species in four different groups of locomotor habits: arboreal, scansorial, semifossorial and terrestrial, following [50]. Using *δ*^13^C and *δ*^15^N, we calculate the standard ellipses (SEA_*C*_ and SEA_*B*_) for each group of locomotor habit and statistically compared the size and overlap between groups in each sampled site and for all sites polled together.

To investigate the role of body size in niche equalities mediating isotopic space partitioning, we used the mean body mass of each species to calculate the Euclidean distances between i) all pairs of species within the same group (rodents or marsupials) for each site and ii) within the same group and locomotor habit for each site, which generated matrix of species pairwise body mass distances. Then, we calculated Euclidean distances of pairwise isotopic ratios among species, which represents a measure of interspecific isotopic niche differences. If there are isotopic niche shifts related with body mass, we would expect that more similar body mass species have more similar isotopic ratios. Then, we would expect the matrix of body mass differences to be positively correlated with the matrix of pairwise isotopic differences. We tested for the correlation between matrices with a Mantel test with 999 simulations. We also ran this analysis for all sites, using all sampled species. We performed statistical analysis in R version 3.1.1 [51] using the packages *siar* and *ade4* [49].

We cannot ran mixing models to estimate proportions of assimilated resources in consumers diet because we acknowledge that, although we collected a substantial number of potential food resources, we do not sampled all the sources of small mammals the field, particularly plants with C4 metabolism and small vertebrates. This is an implicit assumption of mixing models [52] and is particularly challenging in highly diverse biomes with complex trophic networks, as the Atlantic rainforest.

## Results

### Food Resources

We collected 209 samples of four main potential food items for small mammals and categorize them into four source categories: animal prey (spiders, ants, worms, crickets, termites and beetles), leaves, seeds/fruits and fungi (S1 Table). We polled in a single category seeds and fruit pulp because we do not find statistical differences between in the isotopic signature for both *δ*^13^C and *δ*^15^N. Leaves showed a wide range of *δ*^13^C values, because C3, C4 and CAM plants were included in our samples.

### Marsupials vs. Rodents’ isotopic niche

We performed stable isotope analyses using hair samples from 253 individuals of 22 species of small mammals, being 13 rodents and nine marsupials (Table 1). The number of individuals sampled varied from one to 77 (*Euryoryzomys russatus*), which reflect their abundance in the study areas [38, 39]. We acknowledge that for some species only few individuals were captured, however we opted not to remove these species from analyzes and represent the whole local community, not just the most abundant ones.

**Table 1:** Number of captured individuals, mean body mass (g), *δ*^13^C (‰ PDB) and *δ*^15^N (‰ Air) values and locomotor habit (Sca = scansorial, Arb = arboreal, Ter = terrestrial and Sem = semifossorial) of the 22 small mammal species (nine marsupials and 13 rodents) in the Atlantic forest areas.

Taxa	N	Body mass (g)	δ^13^C		δ^15^N		Locomotor habit
			Average	SD	Average	SD	
**Didelphimorphia**							
**Didelphidae**							
*Didelphis aurita*	6	1078.50	-26.78	0.84	5.58	1.50	Sca
*Gracilinanus microtarsus*	2	18.25	-27.56	0.40	2.89	0.46	Arb
*Marmosops incanus*	12	50.46	-26.99	0.78	5.29	0.90	Sca
*Metachirus nudicaudatus*	6	331.67	-26.31	0.51	5.77	0.59	Ter
*Monodelphis americana*	2	22.00	-27.28	0.30	5.81	0.30	Ter
*Monodelphis iheringii*	1	11.50	-27.10	-	7.04	-	Ter
*Monodelphis scalops*	4	43.25	-27.01	0.27	5.21	1.00	Ter
*Monodelphis* sp.	1	10.00	-26.96	-	4.63	-	Ter
*Philander frenatus*	17	218.41	-27.06	0.49	5.29	0.58	Sca
**Rodentia**							
**Cricetidae**							
*Akodon cursor*	4	29.25	-26.52	1.18	5.75	2.05	Ter
*Akodon montensis*	18	29.00	-24.76	5.44	4.93	1.40	Ter
*Blarinomys breviceps*	1	20.00	-26.09	-	7.37	-	Sem
*Brucepattersonius soricinus*	7	31.36	-23.90	3.95	7.27	1.05	Sem
*Delomys dorsalis*	1	68.00	-29.11	-	2.52	-	Ter
*Delomys sublineatus*	2	49.50	-26.97	0.53	2.11	2.14	Ter
*Euryoryzomys russatus*	77	71.54	-27.66	0.90	2.95	1.03	Ter
*Juliomys pictipes*	4	8.13	-27.82	1.10	5.04	1.94	Arb
*Necromys lasiurus*	5	28.90	-13.82	1.61	3.62	1.35	Ter
*Oligoryzomys nigripes*	6	18.75	-16.91	2.86	3.76	0.81	Sca
*Sooretamys angouya*	3	122.67	-27.72	0.10	0.37	0.77	Ter
*Thaptomys nigrita*	30	21.27	-26.44	0.95	5.21	0.95	Ter
**Echimyidae**							
*Trinomys iheringi*	44	204.03	-27.98	1.00	2.29	0.86	Ter

The isotopic data were defined in delta notation (*δ*) and reported in parts per mil (‰) of international standards.

**Fig 2:**
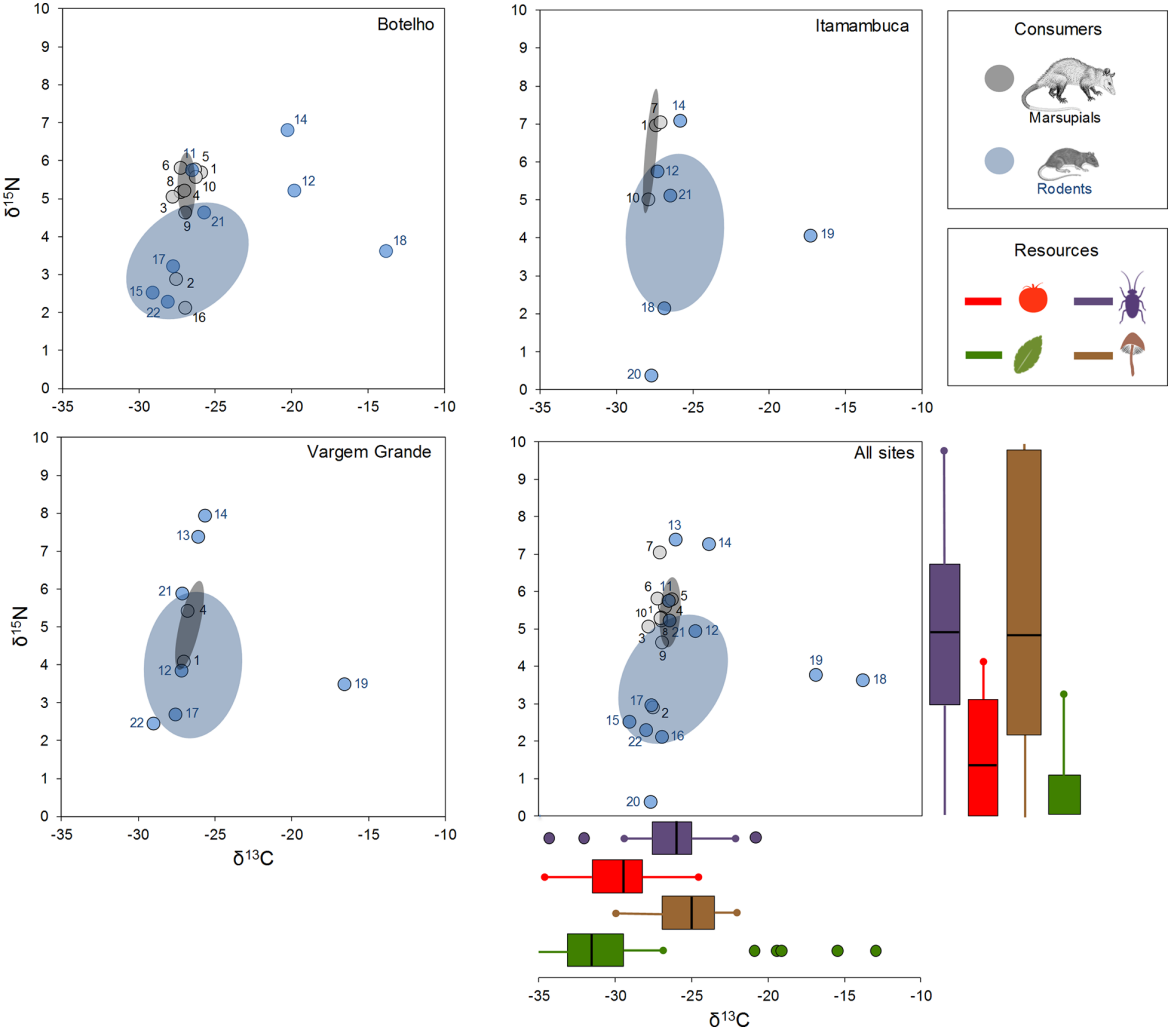
Isotope plot depicting means isotope values (*δ*^15^N and *δ*^13^C) of rodent (gray dots) and marsupial (blue dots) species from Atlantic rainforests, with respective standard isotope ellipses, for each studied site and all sites polled together. Isotope values were corrected downward 2.7‰ (for *δ*^15^N) and 2.4‰ (for *δ*^13^C) to account for trophic enrichment. Isotope values of the potential food resources are represented in boxplots. Species code: 1 = *Didelphis aurita*; 2 = *Gracilinanus microtarsus*; 3 = *Juliomys pictipes*; 4 = *Marmosops incanus*; 5 = *Metachirus nudicaudatus*; 6 = *Monodelphis Americana*; 7 = *Monodelphis iheringi*; 8 = *Monodelphis scalops*; 9 = *Monodelphis* sp.; 10 = *Philander frenatus*; 11 = *Akodon cursor*; 12 = *Akodon montensis*; 13 = *Blarinomys breviceps*; 14 = *Brucepattersonius soricinus*; 15 = *Delomys dorsalis*; 16 = *Delomys sublineatus*; 17 = *Euryoryzomys russatus*; 18 = *Necromys lasiurus*; 19 = *Oligoryzomys nigripes*; 20 = *Sooretamys angouya*; 21 = *Thaptomys nigrita*; 22 = *Trinomys iheringi*.

Stable isotope values of individuals ranged between -0.35‰ and 8.53‰ for *δ*^15^N and between -30.63‰ and -12.37‰ for *δ*^13^C. The nine marsupial species showed very similar *δ*^13^C values (ranging from a minimum of -27.56‰ for *G*. *microtarsus* to a maximum of -26.31‰ for *Metachirus nudicaudatus*, mean values), whereas species of rodents have a significantly broader *δ*^13^C range (from -29.11‰ for *Delomys dorsalis* to -13.82‰ for *Necromys lasiurus*). Particularly, two rodents, *N*. *lasiurus* and *O*. *nigripes*, presented relatively higher *δ*^13^C signatures (Fig 2). In relation to *δ*^15^N, the community of small mammals presented a broad range of values, from 0.37‰ for *Sooretamys angouya* to 7.37‰ for *Blarinomys breviceps*. As observed to *δ*^13^C, rodents showed a wider range of *δ*^15^N than marsupials (Fig 2), suggesting a larger diversification in trophic levels within rodents’ species. Two rodents, *B*. *breviceps* and *Brucepattersonius soricinus*, and one marsupial *Monodelphis iheringii*, presented relatively high *δ*^15^N signatures, which suggests the importance of animal prey and fungi as food sources to these species (Fig 2).

Rodents and marsupials showed clear differences in isotopic niche in all studied sites (Fig 2). The isotopic niche space occupied by rodent species was significantly higher than for marsupials in all studied areas (Table 2, Fig 2). The percentage of the isotopic niche space of marsupials overlapped with the isotopic niche of rodents varied between 12.87 to 94.75%, while only 1.21 to 13.15% of the isotopic niche space occupied by the latter is overlapped with the marsupials’ niche space (Fig 2). In summary, consistently in all studied communities and considering all sites/species polled together, rodents presented larger and less overlapped isotopic niche space in relation to marsupials.

**Table 2:** Richness, comparison between the areas of the isotopic niche space (SEA_*C*_) occupied by rodents vs. marsupials, and percentage of the niche overlapped with the other group for marsupials and rodents in three sites of the Atlantic forest.

Site	Richness	SEA_*C*_ (‰^2^)			% Marsupials' niche overlapped with rodents' niche	% Rodents' niche overlapped with marsupials' niche
		Rodents	Marsupials	p-value		
Botelho	18	17.28	1.59	<0.001	12.87	1.21
Itamambuca	10	20.11	0.49	<0.001	31.43	1.5
Vargem Grande	9	28.3	2.34	<0.001	94.75	13.15
All sites	22	18.86	1.97	<0.001	46.77	4.95

The isotopic data were defined in delta notation (*δ*) and reported in parts per mil (‰) of international standards.

### Effects of locomotor habit and body size on isotopic niche

**Fig 3:**
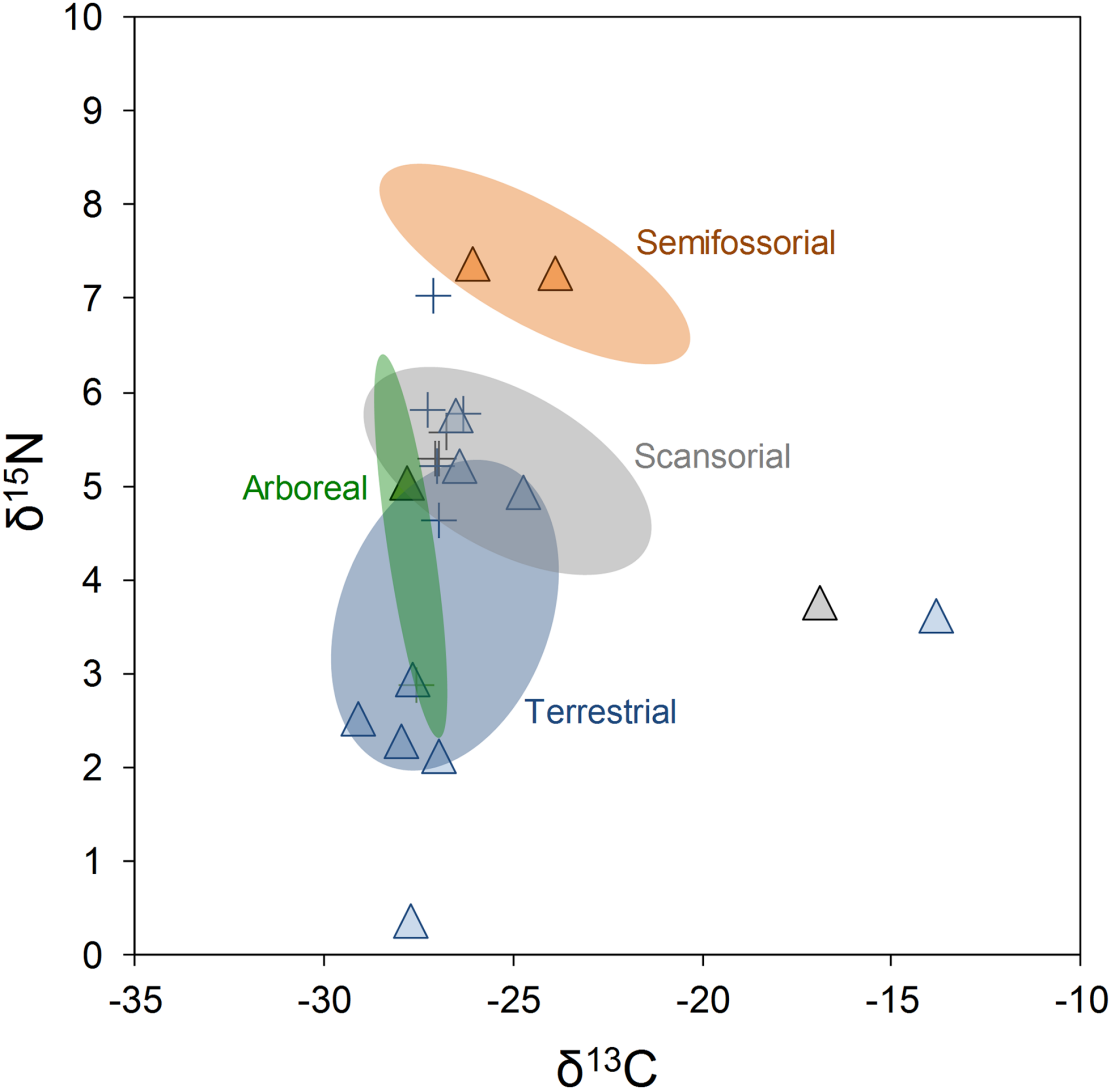
Standard isotope ellipses (SEA_*C*_) from different groups of locomotor habit in communities of small mammals in the Atlantic forest.

Terrestrial species showed larger standard ellipse area than other groups of locomotor habit (p < 0.001, Fig 3), indicating that this group occupies a bigger niche in isotopic space than the others. The standard ellipse area of semifossorial and scansorial was similar in magnitude, although both present no overlap, and the arboreal group—represented by only two species—occupied the lower isotopic niche space (Table 3, Fig 3). We observed a relatively low isotopic niche space overlap between locomotor habit groups, particularly between semifossorial and the other groups (Table 3, Fig 3), suggesting that the use of different forest strata results in distinctions in isotopic niches. Although the average body mass varied largely among studied species (from 8.13 g for *Juliomys pictipes* to 1078.5 g for *Didelphis aurita*, Table 1), the correlation between body mass and isotopic niche differences was not significant in all statistical comparisons: for all species (Mantel; r = -0.15, p = 0.96), ii) for species within the same group and site (all p-values > 0.23) and iii) for species within the same group, site and locomotor habit (all p-values > 0.36), indicating that isotopic niche partitioning is not mediated by body-size interspecific variations.

**Table 3:** Richness, area occupied by the isotopic niche space (SEA_*C*_) and percentage of isotopic niche space overlap between locomotor habit groups of small mammals three sites of the Atlantic forest.

Locomotor habit	Richness	SEA_*C*_ (‰^2^)	% of isotopic niche space overlapped with			
			Terrestrial	Scansorial	Arboreal	Semifossorial
Terrestrial	14	15.11	*100*	18.29	17.28	0
Scansorial	4	10.97	25.17	*100*	9.77	0
Arboreal	2	3.82	68.25		*100*	0
Semifossorial	2	8.42	0	0	0	*100*

The isotopic data were defined in delta notation (*δ*) and reported in parts per mil (‰) of international standards.

## Discussion

The use of stable isotopes revealed distinct isotopic niche patterns among rodents and marsupials that inhabit the largest continuous remnant in Atlantic rainforest in Brazil. Interestingly, we found that rodents and marsupials notably have distinct trophic niches, both in area and the degree of overlap. Marsupials’ isotopic niche space corresponds to only a small subset of the wide isotopic niche space occupied by rodent species, which have a large diversification among species in isotopic space. We also found that interspecific differences in locomotor habits, but not in body mass, are associated with divergences in the position of the isotopic niche space. This pattern indicates that use of distinct forest strata (e.g. ground, understory) may constitute a mechanism of isotopic niche partitioning in small mammals communities, potentially favoring coexistence.

We observed a range of *δ*^13^C (15.3‰) notably broader than the observed in other small mammals communities in tropical and temperate regions [16, 53, 54]. This confirms the complexity of Atlantic forest trophic interactions, indicating that small mammals in this biome—especially rodent species—rely on multiple and diverse basal food sources. Most of the species of rodents present *δ*^13^C values between -30 and -25‰, indicating that plants with C3 metabolism are important basal sources their trophic chains. However, some rodents–particularly of *N*. *lasiurus* and *O*. *nigripes*–showed higher *δ*^13^C values, suggesting that another food sources may be important to these species. We collected potential plant food resources collected with high *δ*^13^C values (between -21.13‰ to -13.14, Fig 1), which likely have CAM metabolism, such as bromeliads and epiphytic cacti (*Rhipsalis* spp.) [55]. However, CAM and C4 metabolism plants may have similar *δ*^13^C signatures [43], therefore we could not tease apart the major carbon source of 18 individuals with observed with *δ*^13^C values, mainly because we did not sampled many C4 potential sources in the forest. In this sense, the rodent species with higher values of *δ*^13^C, *N*. *lasiurus* (five individuals all males) and *O*. *nigripes* (three males and three females), likely have C4 and/or CAM forest plants as important food sources. It is possible that these individuals feed on treefall gaps and forest edges or, particularly the males, are immigrants from neighboring open areas. Although our capture plots were distant more than one kilometer from open areas, it is know that males of *N*. *lasiurus* and *Oligoryzomys* spp. can move long distances [56]. Certainly, future studies using mixing models and considering more potential food sources may help to clarify this issue.

Considering an average trophic enrichment of ~2.7‰ per trophic level [57] and the observed range of *δ*^15^N (7‰), the small mammal community of Atlantic forest encompasses two or up to three trophic levels, a trophic structure similar to others communities of small mammals in tropical regions [16, 53]. However, all the extreme values of this *δ*^15^N range correspond to isotope values of rodent species. Marsupials were concentrated in a relatively small and high position on the food chain (Fig 2), likely relying on sources with relatively high trophic levels (e.g. fungi, invertebrates, small vertebrates). Only one marsupial species, *Gracilinanus microtarsus*, presented relatively lower values of *δ*^15^N (2.89‰), suggesting a diet predominantly based on C3 plant material (probably fruits). Although we only captured one individual of *G*. *microtarsus*, our results contracts with other studies that consider this species mostly insectivore in fragmented forest [58] and Cerrado areas [59]. Interestingly, marsupials are also highly concentrated in a small subset of *δ*^13^C axis, indicating that theses species rely in similar food sources, likely derived from food chains based on C3 plants. These results differ from the previous classic dietary studies, which considered the didelphid marsupials as generalists and “omnivorous”, consuming a wide range of different fruits, invertebrates and small vertebrates [60–62]. This ‘isotopic niche packing’ of marsupials in relation to rodents is a promising area of study and can bring new insights to understand how these different lineages have shared resources along its history of coexistence in Neotropics.

Rodents were more plastic in their use of food resources with species distributed in up to three trophic levels. Typically considered frugivore-granivore species (*Sooretamys angouya*, *Delomys sublineatus*, *Trinomys iheringi*, *Delomys dorsalis*, *Euryoryzomys russatus*, [23]; [17]) composed the first trophic level, while species considered omnivorous in classic dietary studies are at the second trophic level (Fig 2). Interestingly, there is a clear distinction in carbon primary sources within this second trophic level. Some species are extremely enriched in *δ* ^13^C (i.e. *N*. *lasiurus* and *O*. *nigripes*), whereas the others are *δ* ^13^C depleted (e.g. *A*. *montensis* and *T*. *nigrita*). The latter pair of species share ecological characteristics regarding body size, diet and vertical use of space [21], but differ in their period of activity: while *T*. *nigrita* has diurnal habits, *A*. *montanensis* has peaks of activity during the twilight. The activity period is an important dimension in resource partitioning among sympatric species mainly if they overlap in other niche axis and occur in high abundance within a community [63]. The third level of the community encompasses the *Blarinomys breviceps* and *Brucepattersonius soricinus*, likely presenting an insectivore diet. Consistent with these results, [21] found that the stomach contents of *B*. *soricinus* included primarily arthropods.

Contrary to the long-standing expectation that body size is associated with niche partitioning—once species with different body sizes might be able to explore and select preys of varied sizes and types [12, 64], we found that differences in interspecific differences in average body mass were not related to divergence in isotopic values. Interestingly, this pattern is consistent both in whole-community and within coexisting species of the same group sharing the same locomotor habit. Although the hypothesis of niche segregation based on body-size received wide support for various taxonomic groups [14], including desert rodents [65], for others body size was insufficient to explain niche differentiation [66]. Our results indicate that body size alone cannot explain coexistence of small mammals in the Atlantic forest, nor the resource partitioning within groups of species with the same habit of mobility, contradicting classical proposals of community assembly in small mammals [67]. We propose that other mechanisms, particularly the differential use of forest strata, can describe better interspecific niche differentiation. For instance, despite the remarkable difference in body mass between the marsupials *Didelphis aurita* (mean body mass = 1078.5 g) and *Monodelphis scalops* (mean body mass = 43.25 g) both occupied similar positions in isotopic niche space, but forage in different strata, suggesting the effect of vertical spatial segregation between this species.

The observed isotopic niche segregation associated with locomotor habit can be a result of the vertical variation in isotopic ratios of sources available along forest strata gradients (e.g. canopy effect, [68]). For instance, semifossorial species was *δ*^15^N enriched, suggesting a higher consumption of invertebrates and fungi, which are abundant in soil and litter in Atlantic rainforest. In this sense, the use of specific forest strata might determine the energy sources and nutritional quality of resources available to small rodents. As the use of given foraging strata can be modulated by interspecific competition in small rodents [69], possibly species can plastically adjust their microhabitat use according to the intensity of local interspecific competition. Although this is a hypothesis that still needs evidence, it would explain the variation in the use of microhabitats by small mammals between Atlantic forest sites, such as the dominance of marsupials in canopy in given sites, but not in others [70].

In conclusion, we found that the small mammal community of Atlantic rainforest relies on diverse basal trophic sources and is structured in up to three trophic levels. We also observed that interspecific differences in locomotor habit, but no body mass, constitute a driver of isotopic niche partitioning. Considering the worrying conservation scenario of Atlantic rainforests [35], our results emphasize that anthropogenic impacts can threat the trophic dynamics of small mammals communities [71, 72]. Destruction of habitat and fragmentation simplify the vertical structure of ecosystems (e.g. decreased litter, suppression of understory) and can collapse groups of basal trophic resources (e.g. human-modified ecosystems dominated by C4 species), which might constrain the partitioning of isotopic niche by species and collapse the diverse small mammal communities in Atlantic forests [37].

## Supporting Information

S1 Table Stable isotopes of rodent and marsupial species in each study areas in the Brazilian Atlantic forest (Galetti_Plos_SupplementaryMaterial.xlsx). (XLSX)
